# Habsburg Austria: Experiments in Non-Territorial Autonomy

**DOI:** 10.1080/17449057.2015.1101838

**Published:** 2015-12-21

**Authors:** Börries Kuzmany

**Affiliations:** ^a^ Austrian Academy of Sciences, Austria

## Abstract

In the early twentieth century, three provinces of the Austrian half of the Habsburg Empire enacted national compromises in their legislation that had elements of non-territorial autonomy provisions. Czech and German politicians in Moravia reached an agreement in 1905. In the heavily mixed Bukovina, Romanian, Ukrainian, German, Jewish and Polish representatives agreed on a new provincial constitution in 1909. Last but not least, Polish and Ukrainian nationalists compromised in spring 1914, just a few months before the outbreak of the First World War vitiated the new provisions. Even though the provisions of these agreements varied substantially, new electoral laws introducing national registers were at their heart. These were designed to ensure a fairer representation of national groups in the provincial assemblies and to keep national agitation out of electoral campaigns. The earliest compromise in Moravia went furthest in consociational power sharing. However, the national bodies within the provincial assembly had no right to tax their respective national communities, and the provisions of the provincial constitutions kept the non-nationally defined nobility as an important counterbalance. The compromises in Bukovina and Galicia, even if they categorised *all* inhabitants nationally, contented themselves with even less autonomous agency for the national bodies in the provincial assemblies and rather emphasised the symbolic elements of national autonomy. The non-territorial approach in all three crownlands, however, was an instrument to reorganise multi-ethnic provinces that increasingly became the model for national compromises in other Austrian provinces.

## Introduction

The Habsburg Monarchy was not only a multi-national empire, it was also a *monarchia composita*—an assembly of historical provinces called crownlands. Despite its federal structure, its central institutions (government and administration) had been very strong ever since the centralising efforts of the enlightened absolutist rulers of the second half of the eighteenth century. The most significant change in the country's structure was the Austro-Hungarian Compromise in 1867 when the Empire, up to then a relatively centralised state, split into two formally equal entities: the Kingdom of Hungary, also referred to as *Transleithania* (a reference to the little river Leitha separating the two parts), and the Austrian Empire, also referred to as *Cisleithania*.^1^ This was essentially a confederal solution, leaving only the head of state, the currency, customs regulation, foreign policy and the army in common hands; each entity possessed its own parliament, government and administration. The reality and self-perception of the two parts also strongly differed from each other. Hungary became a unitary state recognising a single political Hungarian nation, even though Hungarian speakers did not even account for half of Transleithania's population. The Austrian part, on the other hand, continued to perceive itself as a compound monarchy and a multi-national state. Owing to a century of German language hegemony, German clearly had a dominant position in central state and provincial administration, even though Austria, legally, did not have an official language, but recognised nine customary languages. Still, Cisleithania disposed of a very powerful central government and central bureaucracy; and the legislative power of the provincial diets was rather limited compared to the importance of the lower house of the central parliament in Vienna, the *Reichsrat* (Imperial Council).^2^

This article concentrates on the Austrian part of the Empire, its struggle to deal with its multi-national structure and its innovative approaches to the management of ethnic conflict. The article first sketches the ethno-national landscape and its conflict lines as well as the legal preconditions for nationalities policy. Then it analyses provincial compromises enacted in the early twentieth century in the provinces of Moravia, Bukovina and Galicia and provides an overview of developments in Bohemia and Bosnia-Herzegovina. Even though these agreements differed considerably in the motivations of those who were party to them, in their mechanics and in their ultimate outcomes, they all had a sort of non-territorial component. In the concluding section, the article discusses the influence these models had on the central state's approaches to the reduction of national conflict.

## National Conflicts and Legal Empowerment for Ethno-national Groups

National conflicts in Cisleithania appeared at local, provincial and state levels. The stages on which they were fought out were the political representative bodies (municipal council, provincial diet, central parliament), the courts, the schools and school boards, and, increasingly, the public space. Key elements of the national conflicts included language issues and the struggle to widen or to limit language use within the municipal and provincial administration and jurisdiction, as well as at all levels of education. Thus, nationalists on all sides frequently used language data to support their claims and complaints. These language data were provided by the Austrian Central Bureau of Statistics based on a population census every 10 years. From an early stage, these censuses, their criteria and their methods were hotly disputed by national activists. The Bureau of Statistics did not collect data on citizens’ ethnic affiliation or mother tongue, but only on their language of daily use. This clearly introduced a bias in favour of Cisleithania's lingua franca, German, but also favoured more socially prestigious languages such as Polish in Galicia and Italian in the coastal provinces (Brix, 1982b, pp. 30–35).

In particular, nationalists were often unhappy with the strong linguistic fluctuations between one census and the next, 10 years later, especially when they occurred at the embattled language borders. These fluctuations were unlikely to have resulted from migration, but rather testify to ordinary people's pragmatic, ignorant or reluctant approach towards linguistic self-identification (Judson, 2006; Judson & Zahra, 2012; Zahra, 2008; Stourzh, 2011). Even though the census data intended to be descriptive, they were highly normative in de facto defining national groups, not least because it was not possible to indicate two or more languages of daily use, and every Cisleithanian citizen had to be classified according to the nine customary languages of the Austrian part of the Empire. Although these statistical data are not unproblematic, it may be helpful to report the linguistic distribution in Austria's 17 provinces according to the censuses of 1890 and 1910 (see Table 1).

**Table 1.** Language of daily use, Austria, 1910 and 1890

Language	Speakers 1910	Percentage 1910	Percentage 1890
German	9,950,266	35.6	36.0
Bohemian–Moravian–Slovak	6,435,983	23.0	23.3
Polish	4,967,984	17.8	15.8
Ruthenian	3,518,854	12.6	13.2
Slovenian	1,252,940	4.9	5.0
Italian-Ladin	783,334	2.8	2.9
Serbo-Croatian	768,422	2.6	2.9
Romanian	275,115	1.0	0.9
Hungarian	10,974	0.0	0.0
Total	27,963,872	100.0	100.0

*Note*: The table refers to Austrian citizens only, and indicates the denominations used for the languages at that time. Figures for Hungarian speakers (0.04% of the population in 1910 and 1890) refer only to those who were Austrian citizens, who almost entirely lived in Bukovina, the only region in Cisleithania that regarded Hungarian as a customary language. Many more Hungarian speakers actually lived in Cisleithania, especially in Vienna, but they were Hungarian and not Austrian citizens and, therefore, were not counted in the census.

*Source*: Austria, 1914, pp. 59, 61.

The key legal document behind most of the national conflicts was article 19 of the Basic Law on the General Rights of Citizens, part of the Austrian Constitution of 21 December 1867. This proclaimed the equality of all peoples of Cisleithania, but commingled ethnicity, nationality and language. A similar provision had already been discussed during the Habsburg Empire's first but brief constitutional period between 1848 and 1849. Ever since then, the equality of peoples developed into a sort of a guiding constitutional principle, if with uneven application. Conflicting parties recurrently referred to the three clauses of this 1867 law and periodically alleged infringements of their rights in Cisleithania's supreme judicial institutions, the Imperial Court of Justice (*Reichsgericht*) and the Administrative Court of Justice (*Verwaltungsgerichtshof*). This shows that the struggle for national rights was not only conceived as a political issue but also as a legal matter within the Austrian legal system. As article 19 of the basic law put it,

All peoples [*Volksstämme*] of the state are equal in their rights, and each people has the inviolable right to maintain and to cultivate its nationality [*Nationalität*] and language.

For all languages whose use is customary in the land, the state recognises equality of rights in schools, public offices, and public life.

In those crownlands inhabited by more than one people, public institutions of education shall enable each of the peoples to be educated in its language, without being compelled to learn a second language of the land.^3^

All three sentences gave rise to problematic questions. The first clause does not define a ‘people’, but at the same time endows ‘peoples’ with specific rights. The second clause neither specifies the necessary percentage for a language to be considered a customary language of the land, nor whether to take into account the category ‘mother tongue’ or the category ‘language of daily use’. Finally, the third sentence is an invitation to monolingualism, ignoring the need of mutual communication in order to assure peaceful coexistence. Thus, article 19 gave rise to as many questions as it purported to resolve, and its vague formulation left its intention unclear. Legal scholars discussed intensively whether the law was a broad commitment or promise that would need further implementing rules, or whether it was an actually applicable law. The latter opinion prevailed, and the supreme courts applied article 19 in more than 315 verdicts. Not all decisions were in favour of the claimants; still, this article was crucial for the empowerment of nationalist movements (King, 2010, especially chapters II and III; Stourzh, 1985, pp. 11–12).

The hotspots of national conflict were the crownlands of Galicia (Polish versus Ruthenian^4^ nationalists), Styria and Carinthia (German versus Slovenian), Tyrol (German versus Italian) and the Austrian Littoral consisting of the provinces of Gorizia, Trieste and Istria (Italian versus Slovenian and Croat nationalists). The most ardent national battles, however, occurred between Cisleithania's two largest groups, the Austro-German and the Czech nationalists. Even though they also fought over representation in the central parliament, they especially struggled for dominance in the Bohemian lands, which comprised the crownlands of Bohemia, Moravia and (Austrian) Silesia. While the provincial civil services operated bilingually when dealing with ordinary citizens, the internal language of administration continued to be German only. As Czech civil servants were far more likely to be bilingual, they would have gained more and better positions in public administration if the entire crownland were to be considered bilingual. Finally, in 1897 Czech politicians reached their goal, and the Austrian prime minister Kazimierz Badeni decreed that within four years any Bohemian and Moravian civil servant would have to be able to work in both languages. What followed was the hardest political crisis in late imperial Austria. A wave of German nationalist outrage swept the public sphere and the parliament and finally led to Badeni's resignation and the withdrawal of his decrees (Kořalka, 1991, pp. 146–159, Sutter, 1960).

Nevertheless, the Bohemian lands were not only the region of the most virulent national conflicts, but also the location of the most innovative approaches to their resolution. The provincial constitution, adopted by the Bohemian Diet in 1871, would have provided for strong elements of national autonomy based on the personal and not on the territorial principle. However, the emperor never gave formal legal effect to this so-called Bohemian Compromise due to the fierce opposition of Bohemia's German political representatives. The only provision of the 1871 Bohemian constitution that eventually was implemented two years later was the introduction of nationally segregated school boards in those towns that had both Czech and German schools. The Bohemian law now proclaimed that only members of the respective nationality were allowed to be part of these school boards—thus for the first time introducing the national–personal principle. Not surprisingly, it was not always easy to clearly determine a school inspector's national affiliation. After 1873, the question of how to define a nationality or an ethnic group, and how to determine membership in it, became a legal problem. For decades, Austrian legal practice accepted in cases of doubt a person's individual national declaration instead of relying on quasi-objective criteria such as language or descent (King, 2013; see also Stourzh, 1994, which summarises in English some parts of Stourzh, 1985).

Also closely entangled with the Bohemian lands was the Social Democratic politician Karl Renner, who was born into a German-speaking family in Moravia. In 1899 and 1902, Renner developed a detailed plan to reorganise the Habsburg Empire, as did his ‘Austro-Marxist’ colleague Otto Bauer in 1907. They combined territorial and non-territorial elements in order to secure national self-rule, and interestingly referred to early modern models of jurisdiction based on a person's confessional or feudal status and not their territorial affiliation (Bauer, 2000 [1907]; Renner, 1902; Renner, 2005 [1899]). In the same period, prominent legal scholars such as Bernatzik (1910), Lukas (1908) and Herrnritt (1914) sketched their opinions about the positive and negative features of national–personal autonomy regulations. Based mainly on evidence from cases in the Bohemian lands, they supported the idea of national autonomy, which was the term commonly used at the time, but worried very much about the enforced ascription of nationality on people.

These theoretical and scholarly considerations would require an article on their own. This article, however, confines itself to elements of national–personal autonomy actually realised in one way or another in the Habsburg Empire. Drawn from the experiences of the abortive 1871 Bohemian constitution and the state-paralysing Badeni crisis in 1897, the Austrian government and the emperor refrained from imposing any solution in nationalities issues that was not based on a compromise negotiated between the political representatives of the respective nations.

## The Compromise in Moravia

**Figure 1.**
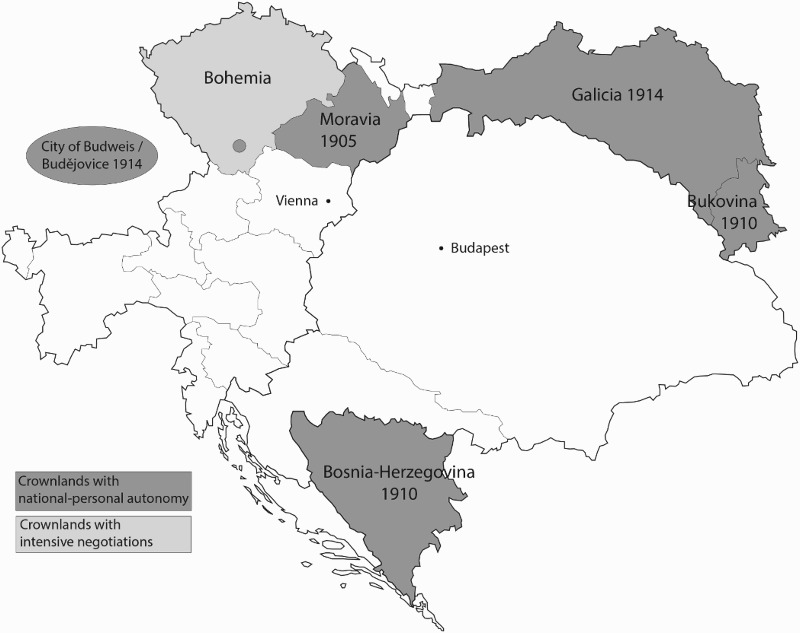
Austrian crownlands that enjoyed a degree of non-territorial regulation *Source*: prepared by author©.

The Margravate of Moravia was the first crownland that reached a compromise partly based on non-territorial principles (for its location, and that of the other crownlands discussed here, see Figure 1). After long negotiations, some reaching back into the 1890s, the provincial diet passed four basic laws in November 1905. These laws introduced a new structure for the diet and the provincial government as well as a new electoral system; furthermore, it touched upon the provincial administration, and on schooling.^5^

Moravia was one of the core Habsburg provinces with close economic and intellectual ties to Prague and Vienna alike. Nationalist tendencies there were less developed than in neighbouring Bohemia, and nationalist activists had to push hard to convince ordinary people that speaking different languages was a key denominator of their identity. In 1900, 71.3% of the 2.6 million Moravians indicated Czech as their language of daily use, whereas 27.9% declared German (Austria, 1914, pp. 59, 61). German speakers had strongholds in the northern and southern bands of the province, but also lived scattered throughout the province, especially in the major cities, and in the provincial capital Brno (German *Brünn*) in particular. For centuries, German was the language of upward social mobility and of the urban bourgeoisie to which newcomers assimilated. As the provincial parliament was meant to represent noble and bourgeois interests, German speakers (who were disproportionately represented among the more privileged social classes) had dominated the provincial assembly ever since the Empire's return to constitutionalism in 1860–1861. However, as the Czech bourgeoisie grew in economic and social importance in the late nineteenth century it increasingly articulated its national demands (Kořalka, 1991, pp. 160–162).

The German absolute majority in the Moravian Diet shrunk from one election to another; in 1902, only 49 of the 100 members of parliament were clearly to be considered German, whereas 45 were Czechs. Only by relying on the six representatives of the non-national aristocratic, but German leaning, *Mittelpartei* (‘middle’ party) could Germans maintain their majority. Most German parties understood that it was only a matter of time before Czech representatives would prevail and have the capacity to out-vote them. They therefore agreed on negotiations to reach a compromise under which they would voluntarily give up their existing majority in exchange for a national veto on key issues. A crucial role in these negotiations was played by the ‘middle’ party, which, in the person of Alfred Freiherr von Skene, also chaired the parliamentary reform committee. The governor of Moravia, who represented the crown and central government, was a fourth participant; the negotiations for this compromise were thus in reality multilateral (Glassl, 1967, pp. 168–169; Luft, 1987, pp. 218–219).

Although many Czech parties joined the cross-national Social Democrats in calling for universal and equal suffrage, the provincial assembly continued as an instrument of the leading social strata, albeit with a fixed national distribution formula. According to the new provincial constitution of 1905, the diet's 151 seats were distributed among four major income-based social electoral classes called curiae. Apart from the supra-national curia of the great landowners, these curiae were subdivided into a Czech and a German section: the urban taxpayers (20 Czech and 20 German), the rural taxpayers (39 Czech and 14 German) and a common curia for all the others (14 Czech and 6 German). Hence, Czechs would gain an assured 73 and Germans an assured 40 representatives. In addition, six nationally unaligned but de facto German or German-Jewish seats were reserved for representatives from the chambers of commerce in Brno and Olomouc (German *Olmütz)* ^6^; and two rather Czech-leaning bishops had *ex officio* mandates. Still, to pass a law in the diet each side would have to win over some representatives from the nationally unaligned 30 representatives of the great landowners. Thus, the great property holders were not only heavily overrepresented in the provincial parliament; they also had the power to tip the scales (Table 2).^7^

**Table 2.** Composition of Moravian Diet, 1905

Curia	Czech	German	Unspecified	Total
Clerical ‘virilists’	–	–	2	2
I. Great Landowners	–	–	30	30
II. Chambers of commerce	–	–	6	6
III. Urban taxpayers	20	20	–	40
IV. Rural taxpayers	39	14	–	53
V. Common curia	14	6	–	20
Total	73	40	38	151

*Note*: The ‘virilist’ bishops were in practice Czech-leaning. The six Chamber of Commerce representatives were de facto German or Jewish.

*Source: Mährisches LGBl. 1906, Nr. 1, Landesordnung*, §§ 3, 3a, 3b, pp. 1–2.

**Figure 2.**
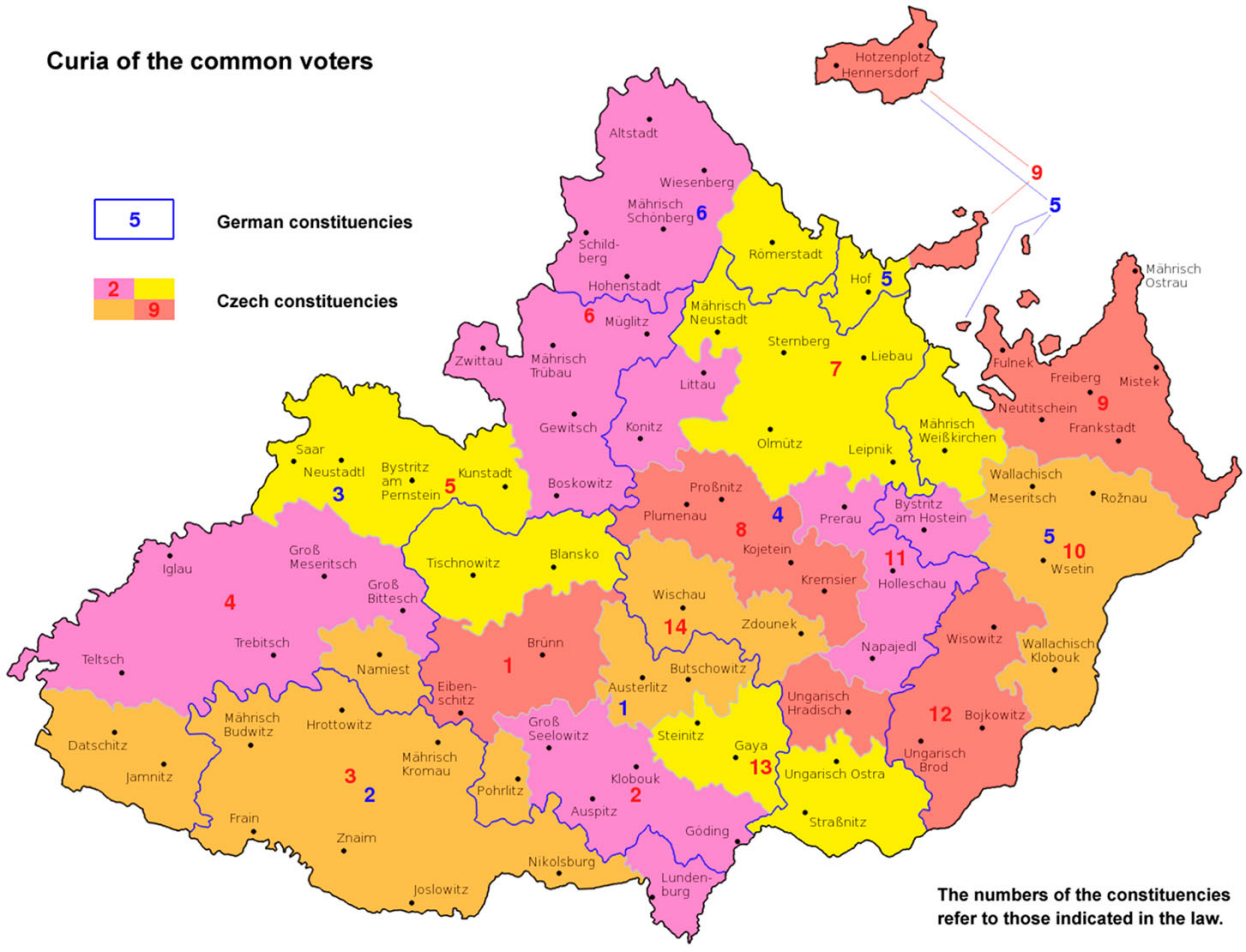
German and Czech constituencies of the curia of common voters for the Moravian provincial elections

*Source*: Wikipedia: Moravian Compromise. User: Amic. Public domain, 30 September 2012. Arranged by author.

In order to guarantee that Czechs would vote for Czechs and Germans for Germans, the 1905 Moravian Compromise introduced national electoral rolls or cadastres, except for the nobility. All mayors were required to register the voters of their municipalities in one of the two national electoral bodies. Once the list was published, everybody on the list was allowed to claim that he, or anybody else on the list, should be transferred to the other register. In cases of dispute, it was the district officer, and not the individual concerned, who had the last say in determining in which cadastre a voter was registered.^8^ Hence, every eligible voter was classified by nationality *and* social curia. Consequently, in total there were seven incongruent layers of electoral districts. Figure 2 illustrates the position by focusing on just two of these seven layers: it shows the 20 constituencies assigned to the fourth curia, that of the common voters. The law did not cut the Moravian map into 20 pieces, rather into 14 Czech constituencies (shaded areas) and six cross-cutting German ones (identified by lines). These electoral districts were smaller in regions where a particular national population was concentrated (for example, in Southern Moravia, with its dense German population). The same system of overlapping Czech-German electoral districts also applied to the curia of the rural taxpayers and the curia of taxpaying urban dwellers. The curia of the land owners, on the other hand, did not have such a national subdivision, but elected its representatives from a land list according to a proportional voting system.

Once the diet assembled for the first time, all members of parliament were required to group themselves into three blocks to elect the eight members of the provincial executive committee (Czech *Zemský výbor*, German *Landesausschuss*), which constituted the provincial government. In this tripartite system the nationally unaligned representatives of the great landowners elected two members, German representatives from all other social curia were assigned two, and the Czech deputies chose four members. Aside from designating the members of the provincial government and of parliamentary commissions, the three blocks did not play any role in the legislative procedure. They had the right neither to levy taxes on their co-nationals nor to pass specific legislation for them. However, the quorum for revision of key elements of the Compromise was high enough to give each of the three blocks a de facto veto in key issues. The constitution required four-fifths of all members of the diet to be in attendance, and support from a two-thirds majority of all delegates present, in respect of the following matters: changing the electoral system, abolishing the national division of the school administration and of the agricultural councils, changing the language of instruction in any schools or closing them, and amending the language of the provincial administration (Glassl, 1967, pp. 215–216). These legal provisions were more consociational than autonomy-oriented in thrust, because they did not introduce separate laws or separate administrations for Czechs and Germans. They did, though, partition the electoral arrangements for the conjoint provincial assembly; this, at least in a symbolic way, gave the impression of autonomous agency of the crownland's nationalities.

The Moravian Compromise's regulations for language in the administration were more than symbolic; they were quite successful in outcome. The entire crownland's administration functioned bilingually with a fixed proportional allocation of Czech and German employees according to the numeric strength of the two peoples.^9^ While the municipalities could choose their working language freely, every individual had the right to communicate with the municipal authorities in his or her own language. If the minority language in a given municipality was less than 20%, the administration could use the translation services of the Moravian government free of charge. In those local communities where the minority language exceeded this percentage, they had to handle the citizen's request on their own.^10^ This was an important accommodation of individual minority rights.

The negotiators of the 1905 Compromise hoped to bring peace to the school system by dividing it in two. In the same way as in Bohemia in 1873, the provincial, district^11^ and local school boards were partitioned into Czech and German sections, though the respective ‘national’ local school boards were elected by the entire municipal council. These boards were allowed to select teachers and headmasters, to choose the teaching materials, to provide a preliminary sketch of the required budget and to inspect the teaching. The boards, however, were not entitled to decide financial matters independently, because the financial means and the obligation to fund the local elementary schools fell to the municipality. According to the Austrian constitution, municipal government had extensive jurisdiction over taxation, and quite often used its power to circumvent its obligations to open minority schools (Glassl, 1967, pp. 215, 217–219; King, 2010, section III). More collective national–personal autonomy was to be found within the secondary schools, which were funded and controlled by the crownland and not the municipalities. In this case, political control over all monolingual educational establishments was exercised solely by the respective co-national members of the provincial government.^12^

In addition, very much on the demand of Czech nationalists, the law obliged public grammar schools to enrol, as a general rule, only those children who had proficiency in the language of instruction.^13^ This provision was designed to prevent Czech-speaking parents from sending their children into German elementary schools that enjoyed a higher prestige and might facilitate social advancement. Accordingly, every autumn the respective Czech and German school boards claimed a number of children who were allegedly registered in the wrong schools. The implementation ordinance for this law, issued by the Austrian education minister Gustav Machet in 1907, allowed and defined several exceptions from the general principles provided for in the law. However, in 1910 the Administrative Court of Justice rather reluctantly overturned this ordinance. It confirmed as lawful the action of a Czech school board that had reclaimed 16 Czech-speaking children from the German elementary school in Uherské Hradiště (German *Ungarisch Hradisch*), arguing that their fathers’ claims to be of German nationality were not justified. This view contradicted the stated right of a person to choose his or her national affiliation freely. This 1910 decision can, therefore, be understood as a paradigm shift in Austrian legal practice. While public authorities earlier had also sometimes investigated people's private lives in order to determine their nationality or that of their children, the Administrative Court of Justice had stuck to its earlier definition of nationality as an individual's free choice. After 1910, however, the Austrian supreme courts increasingly confirmed the view that a person's ethnic belonging can and must be assessed by others in cases of conflict, and that a nation has a right to claim its own members (Burger, 1995, pp. 191–200; Zahra, 2008, pp. 37–45).

## The Compromise in Bukovina

The compromise concluded in Bukovina in 1909 followed the same lines as in Moravia but was more hesitant in strengthening national group rights and in giving autonomous agency to national representatives. Still, the reform of the provincial assembly and the introduction of a new electoral system based on national registers for *all* Bukovinian citizens gave the impression of national autonomy. As Bukovina's ethno-linguistic structure was far more mixed, the provisions of the compromise became extremely complex.^14^

The Duchy of Bukovina was Cisleithania's easternmost province. After its annexation in the late eighteenth century, it was an administrative part of Galicia; it became an independent crownland only in 1849. Owing to several waves of migration, Bukovina was perhaps the Empire's most linguistically mixed province. The population census of 1910 listed five major languages of daily use among Bukovina's approximately 800,000 inhabitants. None had an overall majority: 38.4% spoke Ukrainian, 34.4% Romanian and 21.2% German, with smaller groups speaking Polish (4.6%) and Hungarian (1.3%) (Austria, 1914, p. 61; Scharr, 2010; Stambrook, 2004).

However, the language census ignored three important groups: Jews, Armenians and Lipovans. The roughly 3,000 Lipovans were Russian-speaking Old Believers and were a popular motif in Bukovina's multi-ethnic folklore; they normally indicated Ukrainian as their language of daily use, because Russian was not an officially recognised language in Austria-Hungary. Numerically less significant were the approximately one thousand Armenians, who had largely assimilated earlier to the Polish language. Armenians, however, had a strong position within the landed nobility. The most important group ignored by the language census, though, was the province's approximately 100,000 Jews (13% of all Bukovinians). The Austrian constitution of 1867 did not acknowledge Jews as a nationality but only as religious group. Article 19 of the constitution, discussed above, thus did not apply to them, and neither Yiddish nor Hebrew were recognised languages. Therefore almost all (95%) Bukovinian Jews indicated German as their language of daily use. This was particularly the case for the large assimilated Jewish community in Chernivtsi (German *Czernowitz,* Romanian *Cernăuţi,* Polish *Czerniowce*) with its more than 25,000 members. However, in the smaller towns and villages, Yiddish clearly prevailed (Austria, 1914, Vol. 1, Heft 1, pp. 54, 59, 61; Heft 2, Tabellen, pp. 50, 54–55).

Still, it would be misleading to overstress the importance of the national groups in a period when social status or religion mattered far more: German-speaking Catholics and Protestants, for example, identified differently and mostly inhabited different villages. Ordinary Romanians and Ruthenians, on the other hand, had few conflicts on a daily basis, because they both belonged to the Greek-Orthodox Church. Traditionally, Romanians dominated the higher Orthodox ecclesiastical structures, but the Church's consistory also pursued its own interests, not least because it was by far the province's largest landowner. Other great property owners included the old landed Romanian nobility, Poles and Polish-speaking Armenian Catholics and, increasingly, wealthy Jews. The large majority of Jews, however, were neither proprietors nor wealthy assimilated urban dwellers, but Yiddish-speaking and mostly Hassidic day labourers and petty traders (Scharr, 2011; Stambrook, 2004; Rechter, 2008).

Nationalism did come into play during the last decade of the nineteenth century. However, it was expressed in more moderate and perhaps more conciliatory ways than elsewhere, as none of Bukovina's ethno-confessional groups had a numerical majority in the province. No language would realistically be able to dominate over the others in a new future. Thus, for pragmatic reasons, German continued to be accepted as the crownland's lingua franca in administration and politics. Politicians of all nationalities and social groups understood that at least minimal reform of the province's constitution was necessary and that enlarging political representation was unavoidable. Still, when the negotiations started in 1908 it was also clear that a compromise would not be a full democratisation based on universal equal suffrage, but a sophisticated balance of popular, national and corporate interests (Leslie, 1991, pp. 117–123).

After a year of negotiations, a provincial assembly member of the Romanian Democratic Party, Alexandru de Hurmuzachi (German *Alexander von Hormuzaki*), elaborated a new provincial constitution modelled after the Moravian Compromise. In October 1909, the Diet unanimously passed a new electoral law that would have introduced five different classes of members of parliament: unelected ‘virilists’ (high office holders with ex-officio membership—the archbishop and the university rector), and representatives of four electoral classes (the chamber of commerce, the great landowners, the taxpaying citizens and the general voters). Within these four classes, everybody was originally enlisted in one of five different national rolls: Romanian, Ruthenian, Polish, German or Jewish (Leslie, 1991, pp. 124–125). However, the Ministry of Interior Affairs vetoed the implementation of this electoral law, because it considered the inclusion of a *national* curia for Jews as unconstitutional; it also feared that such a regulation could become a binding precedent for the rest of the Empire. Against the will of the Bukovinian Diet but with the strong support of Austria's assimilated Jewish community, the Ministry forced Germans and Jews into one national register (*Oesterreichische Wochenschrift*, 15.10.1909, pp. 714–715; *Neue Freie Presse*, 26.9.1909, p. 2; Leslie 1991, pp. 137–139).

The final version of the compromise, enacted in May 1910, outlined the German voting districts in such a way as to guarantee in practice a fixed number of Christian and Jewish German representatives without mentioning Jews directly (Rachamimov, 1996, pp. 7–12; Stourzh, 1995, pp. 47–51). In addition, Jews would in practice have two additional mandates via the representatives to be elected from the members of the chamber of commerce. Even though this curia was defined non-nationally, wealthy Jewish merchants so clearly dominated the chamber that these two seats were clearly classifiable as Jewish. The same concealed national affiliation applied to the ‘virilist’ mandates. The legally non-national Orthodox archbishop was always in fact a pro-Romanian conservative; and the rector of Černivci's German university counted as a German, even though the university had seen Jewish rectors as well. The new constitution was, thus, hesitant in openly reducing the entire diet to an ethno-national proportional representation organ. The 63 members of Bukovina's provincial parliament should thus be distributed in principle as shown in Table 3.

**Table 3.** Proposed composition of Bukovkina Diet, 1910

Curia	Romanian	Ruthenian	*Jewish*	German	Polish	*Armenian*	Total
Virilists:							
* *(a) *Archbishop*	1	–	–	–	–	–	1
* *(b) *Rector*	–	–	–	1	–	–	1
I. Great Landowners:							
* *(a) *Orthodox Consistory*	1	–	–	–	–	–	1
* *(b) *Clergymen*	1	1	–	–	–	–	2
* *(c) *Landowners*	4	–	2	–	–	4	10
II. Chamber of Commerce	–	–	2	–	–	–	2
III. Taxpayers	10	10	3	4	1	–	28
IV. Universal	6	6	2	3	1	–	18
Total	23	17	9	8	2	4	63

*Note*: The rector, the archbishop and the great landowners’ representative of the Orthodox Consistory were nationally unaligned and pursued their own interests. However, the rector was rather leaning towards the German and the two others to the Romanian national cause. The figures for Jewish and Armenian members reflect the de facto position; they were formally included, respectively, under the German and Polish headings.

*Source*: *Bukowinisches LGBl*. 1910, Nr. 26: *Landesordnung*: Art. I, § 3, pp. 97–98.

As in the case of the provisions in Moravia, the election of the members of the provincial government and parliamentary commissions proceeded according to the votes of six electoral bodies. These bodies were partly congruent with the social curiae and partly united the co-nationals of different social curiae. Thus, eventually, the provincial government would consist of eight people: three Romanians (one appointed via the clerical representatives), two Ruthenians, one (Armeno-)Pole, one German and one Jew.^15^

On the other hand, unlike in Moravia, all Bukovinians including the landed nobility were registered in national rolls, often called cadastres, not least because in the fourth (universal) curia everybody was enfranchised, even those who were allowed to vote in other curiae. In this respect the Bukovinian Compromise went further in nationalising the entire population than its Moravian predecessor. The national categorisation of all citizens was more difficult. Bukovinian Jews, who had a strongly distinctive but not necessarily national identity, were unhappy at having to join the German register. Some minor groups were also included in other nationalities’ cadastres: Hungarians had to join the Romanian cadastre, Lipovans the Ruthenian and Armenians the Polish one. The Armenian landowners, however, gained representation together with the Poles through a ‘Polish and Armeno-Polish’ electoral body. The local authorities were required to draw up the preliminary national registers and make them public. As in Moravia, everybody on the list was allowed to challenge any other registered person or claim non-registered persons as part of the cadastre; such cases would eventually be decided by the authorities. However, if an individual were to personally delete his name from one national list and sign onto another one, this decision could not be contested by anybody.^16^

In fact, during the first elections according to the new provincial constitution in 1911, approximately 2,000 complaints were filed among the almost 25,000 voters in Černivci, because national labelling proved to be very difficult (Leslie, 1991, pp. 133, 136). The tacit distribution of Christian and Jewish mandates within the German curia also failed to work as planned: The system of double mandate constituencies, where the candidates who ran first and second would both receive a diet seat, was based on unitary national block parties, where all Jews would vote unanimously for a Jewish candidate and all Germans for a German. In 1911, however, there were three candidates campaigning for two seats, two opposing Jews and one German. Eventually, the German candidate ran third, and, therefore, Jews gained both seats in this double mandate constituency. Consequently, Jews eventually held, in total, 10 instead of nine seats in the provincial assembly whereas Germans held only seven instead of eight (Hensellek, 2011). Most national leaders, therefore, saw the whole Compromise more as the beginning of a national autonomy regulation than as the final word in this matter.

The non-territorial provisions of the Bukovinian Compromise and their implementation in the province's realpolitik in general need further investigation. The new provincial constitution did not call for proportional representation of all nationalities in public office as in Moravia; neither did it provide for partition of the provincial school board, and it certainly did not include financial autonomy. However, the law stated at least vaguely that only the respective national members of the provincial government should exercise political control over institutions dedicated to their national group.^17^ The final outcome was primarily a consociational system limiting national autonomy to the symbolic level. The few remaining years of parliamentary work in the provincial assembly up to the First World War prove that the deputies did not consistently make decisions along national lines. The representatives, indeed, formed national parliamentary clubs within the diet, but personal or ideological frictions within them were frequent and ‘inter-ethnic’ alliances a common occurrence.

## The Compromise in Galicia

After many decades of conflict, on 14 February 1914 a compromise between Poles and Ruthenians finally passed the Galician Diet. In addition to a new suffrage law that would allow Ruthenians greater representation in the provincial parliament, this compromise suggested the foundation of a second university in L'viv (Polish *Lwów*, German *Lemberg*) that would operate through the medium of Ukrainian.^18^

The Kingdom of Galicia and Lodomeria, as it was officially called, was Cisleithania's largest and most populous crownland, with eight million inhabitants in 1910. Polish-speaking Roman Catholics were predominant in Western Galicia, whereas in the province's east Ukrainian-speaking Greek Catholics (Uniates) prevailed. Yiddish-speaking Jews lived in both parts of the crownland; only in the larger cities did progressive Jews assimilate linguistically, to German in earlier decades, and to Polish increasingly after the 1880s. While in Western Galicia the Polish-speaking population was socially more diversified, in Eastern Galicia the social dividing lines between Ukrainian-speaking peasants and Polish-speaking landlords largely coincided with the ethno-confessional ones.

As in the rest of the Empire, language statistics were one of the battlefields of national activists. Collecting data on people's language of daily use clearly favoured the Poles, because Polish had been the dominant language in the public sphere ever since the 1870s, not least because the law did not permit identification with Yiddish in the census. Consequently, the Ruthenian national movement preferred to rely on census data reporting religious denomination in accounting for their share of the total population, identifying all Roman Catholics as Poles and all Greek Catholics as Ruthenians. By the early twentieth century, non-Jewish German speakers were not very numerous and mostly lived in either Protestant or Catholic rural settlements (Röskau-Rydel, 2002). The barely 1,400 members of the Armenian Catholic Church had linguistically and culturally assimilated to Polish (Kuzmany, 2008). However, thanks to their archdiocese in L'viv and the importance of quite a few wealthy merchant families, Armenians enjoyed a certain visibility in Galicia. According to the census in 1910, the linguistic and religious distribution was as reported in Table 4.

**Table 4.** Population of Galicia by religion and language, 1910

Religion	Language of daily use			Total (including other)	
	Polish	Ukrainian	German	Number	%
Roman-Catholic	3,616,753	42,822	36,623	3,730,898	46.5
Greek-Catholic	235,328	3,141,029	596	3,379,233	42.1
Jewish	808,327	21,513	25,631	871,804	10.9
Protestant	7,854	834	27,129	37,693	0.5
Armenian-Catholic	1,392	0	0	1,392	0.0
Total (including other)	4,670,167	3,208,025	90,130	8,024,524	100.0
Total (%)	58.2	40.0	1.1	100.0	100.0

*Source*: Austria, 1914, Vol. 1, Heft 2, Tabellen, pp. 50, 54–55; for the number of Armenian-Catholics see Vol. 1, Heft 1, p. 54.

Galicia enjoyed a certain degree of de facto self-rule since the 1870s, mostly in educational, cultural and administrative matters, even though constitutionally it did not enjoy any special status different from that of the other crownlands. Hence, ‘Galician autonomy’ resulted largely from non-interference on the part of the central government (Binder, 2006). Adding to this, it was not the territory of Galicia that benefited from this de facto self-rule but the province's dominant Polish elites. In exchange for the support of the Polish parliamentary group (a powerful collection of mostly aristocratic conservative and rightist liberal representatives, but not including the Peasant Party and the Social Democrats) in the central parliament in Vienna, the Austrian governments allowed them to Polonise the public sphere in Galicia. Most important was the concession that allowed the entire provincial administration to switch from German to Polish and the practice by which the Ministry of Education hardly ever intervened in the activities of the Provincial School Board (German *Landesschulrat*, Polish *Rada Szkolna Krajowa,* Ukrainian *Krajova škil'na rada*). The Board first replaced German by Polish as the language of instruction in almost all Galician secondary and higher education institutions, and later only very reluctantly approved Ukrainian schools, especially gymnasia (high schools).^19^

Although Ruthernians traditionally had few social advancement opportunities apart from clerical careers, a small Ruthenian middle class was beginning to emerge by the late nineteenth century, and increasingly claimed national rights. Talks about a new provincial constitution started in 1905. The Ruthenian National Democrats but also the Polish Peasant Party and the Social Democrats called for the introduction of universal and equal suffrage on the model of that introduced for the Austrian central Parliament in 1907. However, as in Moravia, Bukovina and other crownlands, the Austrian government as well as the regional elites clearly wanted the provincial assembly to be a forum for the representation of corporate interests. So, when intensive negotiations between the Polish parties and the leading Ruthenian representatives began in 1912, the central issue was the distribution of representatives between the national groups. The solution that was found in Galicia was a modified and moderated version of the Moravian and Bukovinian models. After a first draft bill to introduce fairer political representation failed in 1913, in February 1914 the Galician Diet finally approved a new electoral law enfranchising for the first time all male (in some cases also female) Austrian citizens permanently living in Galicia. As mentioned earlier, votes were not equal. Every citizen was assigned to one of six curiae according to their place of residence (town or countryside), their income and/or their social status (Kuzmany, 2013, pp. 133–137).

Even more complicated than the curia system was the assignment of electoral districts to a specific nationality (see Table 5). Very much to the dismay of Zionists and Germans, the law provided only for a Polish and a Ruthenian national roll (Locker, 1914). To be more precise, the de facto Polish register was called the ‘general’ cadastre, and comprised everybody except Ruthenians. This reflects accurately what Galicia represented in the eyes of the Polish establishment: in general Galicia was a Polish land, where a Ruthenian minority happened to live as well. Still, it was not a mere minority register, because *all* Galician citizens were registered. In this respect, Galicia followed the Bukovinian model, whereas in 1905 the Moravian nobility was still considered a non-national element. In Galicia, the local authorities were required to compile a total of 10 voters’ lists: one each for the curia of the chambers of commerce (IV) and the curia of the craft corporations (V), which were organised non-nationally but depended on membership in these institutions; and a Polish and a Ruthenian one for each of the other four curiae (I, II, III and VI). In order to put every voter on the correct list, the local authorities had to take into account their tax records on the one hand, and the language data from the last census on the other. Once the local authorities published all 10 voters’ lists, everybody concerned was entitled to appeal against this assignment.^20^ This turned the once presumably politically neutral collection of data on people's language of daily use in the census into a profession of nationality—an interpretation that national movements had propagated for decades.

**Table 5.** Proposed composition of Galician Diet, 1914

Curia	Polish	Ruthenian	Total
Virilists:			
* *(a) *Clericals*	5	3	8
* *(b) *Academics*	4	1	5
I. Great Landowners	44	1	45
II. Wealthy urban taxpayers	40	6	46
III. All other urban dwellers	9	3	12
IV. Chambers of commerce	5	–	5
V. Craft corporations	2	–	2
VI. Rural population	57	48	105
Total	166	62	228

*Note*: The fourth and fifth curiae were the only ones that were nationally unaligned, because the right to vote depended solely on membership in these organisations; they counted as Polish seats, though. In the sixth curia, wealthy taxpayers had two votes.

*Source*: *Dziennik ustaw dla Galicyi, 1914, Nr. 65: Statut krajowy*, Art. 1, § 3, pp. 169–170; *Ordynacya wyborcza*, § 8, p. 181; and *Dodatek tabelaryczny do ordynacyi wyborczej sejmowej*, pp. 207–219.

Even though the Galician Compromise did not award any national representation to the Jewish population, the way in which the ‘Polish’ constituencies were drawn in the curia of the taxpaying urban dwellers guaranteed at least a certain representation of the Jewish bourgeoisie. Furthermore, among the five representatives of the curia of Galicia's chambers of commerce – which was legally nationally unaligned, but counted as Polish – it is probable that at least three Jewish candidates would have been elected (Kuzmany, 2015). In addition, the Armenians were granted representation via their archbishop, who ‘ex officio’ received one of the nine Polish ‘virilist’ seats; four others would go to Ruthenian ‘virilists’. The set-up of the Galician Diet according to the new provincial constitution and electoral law would, thus, have been as shown in Table 5.

In general, Ruthenians continued to be heavily underrepresented, because they could realistically gain only 27% of the total 228 seats in the Galician Diet. Within the provincial government, their representation would have been even lower. Only two out of eight members would be Ruthenian.^21^ Still, this was already better than the position before the reform, when they accounted for only one out of six members.

The electoral system was cleverly devised to minimise the chance of surprise results, and the ratio of 166 Polish to 62 Ruthenian deputies would not be likely to be violated. It was partly based on a territorial and partly on a personal principle. Whereas all Galicians had to register in either the Ruthenian or the general (and thus in effect Polish) cadastre, not all constituencies were ethnically homogeneous. In the predominantly Polish western part of Galicia, voting districts were territorial; consequently, Ruthenians who had migrated, for example, to Cracow could in practice vote only for Polish candidates. In Eastern Galicia, on the other hand, even the smallest Polish pockets in Ruthenian villages were transferred to the net of Polish constituencies according to the personal principle. In other words, this system fostered assimilation in Western Galicia and strengthened national diversity in Eastern Galicia (Kuzmany, 2013, pp. 132–133).

Undoubtedly, Ruthenian nationalist politicians were not happy with these results. However, they were pragmatic enough to realise that this was the best deal they could get at this point. Most of them saw it as a first step in a new direction; and they expected the foundation of a Ukrainian university and the establishment of more Ukrainian high schools to be a keystone in further strengthening their national case (Binder, 2003). Nevertheless, unlike in Moravia, the Galician school board would not have been split into two sections, and the Ruthenian diet members would have had no autonomous agency in educational or cultural affairs. Thus, even if the Galician Compromise took up some components and symbols of the earlier compromises and applied elements of non-territoriality in the electoral law, it was rather a compilation of Polish concessions towards Ruthenians than a fully developed form of national–personal self-rule.

The Galician Compromise is extremely interesting, even though it was never implemented due to the outbreak of the First World War. Unlike the Germans in Moravia and any of the ethnic groups in Bukovina, the Poles clearly dominated Galicia numerically, economically and politically. Preliminary research suggests that it was public opinion in the Empire favourable to compromise in combination with finely judged political intervention from Vienna that forced the Polish establishment to cede some power to the Ruthenian national movement (Kuzmany, 2013).

## Other Compromises: Bohemia, Budějovice, Bosnia

Inspired by the Moravian and Bukovinian cases, the conflicting parties in Bohemia also tried to negotiate a compromise. Such a settlement would have had to combine some sort of territorial self-administration in the more compactly German districts with non-territorial autonomy elements based on national registers in the entire crownland. Nationalists on both sides had been agitating for decades, and as a result, positions were entrenched across all social groups. Even though the principal modalities of the agreement were negotiated and Vienna and the Bohemian governor pressed hard, neither side was ready to compromise on the details (Seibt, 1987; Waldstein-Wartenberg, 1959). Interethnic cooperation could only be achieved at the local level. In February 1914, only two weeks after the Galician Compromise, the city council in the South Bohemian town of Budějovice (German *Budweis*) agreed to split the German and Czech electorate into autonomous national bodies. This settlement went further than any of the earlier compromises. As in Bukovina and Galicia, it registered all inhabitants according to their nationality but tremendously enlarged the agency of the autonomously elected national representatives. For the first time, they would have received the right to separate national taxation and spending (Brix, 1982a; King, 2002). Even though the Budějovice Compromise was never legally implemented, it demonstrates that the idea of national–personal autonomy developed continuously and could be applied not only at state or provincial level, but also at municipal level.

In 1910, Bosnia-Herzegovina was given a provincial constitution that also resembled the representation model of the Moravian and Bukovinian Compromises. It was, however, constitutionally different from the other provinces. For long part of the Ottoman Empire, since its occupation in 1878 by Austro-Hungarian imperial forces it had been administered by the joint Austro-Hungarian minister of finance;^22^ and every decision needed to be approved by the Austrian and Hungarian governments (but not by the respective parliaments) (Heuberger, 2000). After Bosnia's formal annexation in 1908 (when it was incorporated into imperial structures), the need for its own constitution and legislative body became increasingly obvious. Finally, in February 1910 the emperor decreed a constitution, which ceded limited administrative and legislative autonomy to the province but still required the approval of the Austrian and Hungarian governments.^23^

The electoral law distributed the entire electorate over three social curiae and classified all voters according to their religious denomination in such a way that the Orthodox group would win 31 representatives, Muslims 24, Catholics 16 and Jews one. In addition to these 72 mandates, 20 ‘virilists’ from the clergy, politics, commerce and society would receive *ex officio* seats in the diet. The nine members of the provincial government would consist of four Orthodox members, three Muslims and two Catholics (Imamović, 2006, pp. 200–203, 244–247). Unlike Moravia and Bukovina, Bosnia's electoral law did not have national but rather religious groups at its basis. This was only possible because it was legally a *corpus separatum* and therefore not subordinate to the Cisleithanian constitution that strictly disassociated political rights from religious affiliation. This gave the whole enterprise a rather pre-modern outlook but actually accommodated all sides. Austrian bureaucrats were happy that they did not have to judge who belonged to which nationality but could rely on the ecclesiastical registers. The large Serbo-Croatian-speaking Muslim community and the tiny Sephardic community would have their representatives without reverting to the concealed and complicated formula for Jewish representation in Bukovina and later Galicia. Finally, it satisfied those nationalists who claimed every Orthodox person to be a Serb and considered Catholic to be synonymous with Croat.

## Conclusion

After the turn of the century, we see a rapid growth of interest in the idea of national–personal autonomy in the Habsburg Empire—not necessarily in the fully fledged version envisaged by Austro-Marxist theorists but rather in the pragmatic approach of regional compromises based on consociational and corporate representation. Many political protagonists as well as non-nationalist public opinion leaders seemingly considered it unrealistic to change the territorial setup of the country and to create more ethnically homogeneous provinces. Therefore, they supported non-territorial approaches to soften national conflicts. However, what contemporaries called ‘national autonomy’ was a form of national power sharing dressed up as national self-rule, but amounting to ‘autonomy’ only at the symbolic level, at most.

The general idea behind all these experiments was not to partition the political life of the provinces’ nationalities, but to remove national struggles at least from election campaigns. The new provincial constitutions enacted in Moravia (1905), Bukovina (1909), Bosnia (1910) and Galicia (1914), as well as the new municipal charter in Budějovice (1914), allowed each national group to elect its representatives autonomously. Therefore, they all introduced national or ethno-confessional registers and a fixed number of representatives for each national group. This allowed a fairer but also less flexible national representation in the provincial assemblies and governments, and partly also in the administration of these crownlands.

Applying the principle of national affiliation to all nationalities living in the province, these regulations made no legal distinction between majorities and minorities within the province, but simply tried to reorganise it along multi-ethnic lines. If we look into the matter in detail, however, these regulations were essentially instruments designed to secure national rights for all members of recognised nationalities, even in areas where they were in a minority. Thus, these compromises recognised nationalities as non-territorial agents but hardly granted them autonomous agency. Nowhere did the new constitutions introduce financial autonomy, separate political representative bodies or separate laws for the different ethnic groups. The Bohemian lands appeared poised to move in this direction: the 1914 Budějovice Compromise foresaw national taxation, and in the same year Czech and German politicians were negotiating a revision of the 1905 Moravian Compromise to include budgetary autonomy. But these developments were overtaken by the outbreak of war (Malíř, 2006).

The role of the Viennese central government in these five cases is interesting as well. It strongly approved of all of these negotiations, and insisted that no agreement should be reached without the consent of all national groups concerned (Urbanitsch, 2006, pp. 45–48). It favoured universal male suffrage but, very much to the dismay of the Social Democrats, who sought fairer representation for the working class, the government repeatedly refused to concede equal suffrage. It stated explicitly that it wanted the provincial diets to be representative bodies for corporate and not political interests.^24^

Yet, the degree of intervention varied considerably. In Moravia, the Austrian government remained largely aloof, and eventually accepted provisions, in such areas as schooling, that contradicted Cisleithania's long established liberal handling of national affiliation. In Bukovina, on the other hand, the government bluntly overruled the unanimous wish of the negotiators to introduce a Jewish curia, arguing that this would be unconstitutional and a negative development for the entire state. In Bosnia, in contrast, the government introduced a ready-made constitution based on de facto ethno-confessional registers without consulting local politicians. In Galicia, the Cisleithanian government frequently intervened when the negotiations were in a deadlock, not least because it wanted to pacify this province at a time when tensions with neighbouring Russia were steadily increasing. In the final months before the agreement, the Austrian prime minister, Karl Stürgkh, engaged in almost daily correspondence with the Galician governor, with key politicians and with church dignitaries in order to bring the adversaries together (Kuzmany, 2013, pp. 124–125).

While federalist solutions were discussed as well, in the early 1910s non-territorial concepts were at the top of the agenda in the Habsburg Empire as a mechanism for reducing national conflict. The Emperor himself imposed a cadastre-based model on Bosnia-Herzegovina, the Cisleithanian government strongly pushed the Galician Compromise, and public opinion expected that only a similarly knit compromise could bring peace to Bohemia. More crownlands might have experimented with these ideas in the years to come; however, the outbreak of the First World War brought the further evolution of this approach to an abrupt end. The dissolution of the multi-national empire in 1918 gave way to newly created nation-states that officially dismissed earlier Habsburg experiences with non-territorial autonomy.

## Notes

1. In fact, there was no consensus on what to call the Cisleithanian part. Its official denomination between 1867 and 1915 was ‘The Kingdoms and Lands represented in the Imperial Council’. The term ‘Austrian Empire’ for Cisleithania was legally introduced only in 1915, though this had been widely used ever since 1867.

2. For a good introduction on how the relationship between the central and the provincial administrations was organised in Cisleithania, see Hye (2000).

3. As cited by Wright (1919). This translation was improved and adapted by Jeremy King and the author.

4. I prefer the ethnonym ‘Ruthenian’ over the later ‘Ukrainian’, because it was used until the early twentieth century both in Austrian official terminology and by the people themselves. If referring to the language, though, I use the term ‘Ukrainian’.

5. For general information on the Moravian Compromise, if mostly on the associated political circumstances, see, in English, Kelly (2003). The best explanation of the compromise in English is the as yet unpublished article, King, 2010. For the centenary of the Moravian Compromise, the University of Brno organised a conference; see Fasora, Hanuš, and Malíř (2006). An older but detailed study in German is Glassl (1967). Since he was in charge of actually drafting the law, von Skene's (1910) recollections are also very interesting.

6. In the long run, Czechs might have taken over the seats of the two chambers of commerce because of the growing strength of the Czech commercial elite.

7. 
*Landesgesetz- und Verordnungsblatt für die Markgrafschaft Mähren (Mährisches LGBl). 1906. I. Stück. Nr. 1–4. Gesetze vom 27. November 1905*. Nr. 1, §§ 3, 3a, 3b, pp. 1–2. The German version of the provincial law gazette is available at the ALEX online law collection of the Austrian National Library: http://alex.onb.ac.at/cgi-content/alex?apm=0&aid=lma&datum=1906. See also Glassl (1967, p. 197).

8. 
*Mährisches LGBl*. 1906, Nr. 2, §§ 31, pp. 23, 38–39.

9. 
*Mährisches LGBl*. 1906, Nr. 1, § 32a, p. 8.

10. 
*Mährisches LGBl*. 1906, Nr. 3, §§ 2, 3, pp. 41–42.

11. The district boards were not necessarily congruent with the territory of the political districts, but comprised all schools using one of the two languages in a defined region that could consist of several parts of one or more political districts.

12. 
*Mährisches LGBl*. 1906, Nr. 1, § 32a, p. 8.

13. 
*Mährisches LGBl*. 1906, Nr. 4, Abt. II, § 20, p. 50.

14. There is only one short academic article on the Bukovinian Compromise in English and this concentrates on the Jewish aspect; see Rachamimov (1996). For the very few studies in German, see the excellent article by Leslie (1991). Other introductory studies include Hensellek (2011), and Kotzian (1992). On the development of the Bukovinian Diet before and after the compromise, see Ceauşu (2000).

15. 
*Gesetz- und Verordnungs-Blatt für das Herzogthum Bukowina* (*Bukowinisches LGBl). 1910. XXIII. Stück. Nr. 26*–*27*. *Gesetz vom 26. Mai 1910,* Nr. 26, §§ 10a, 12, pp. 98–100. The German, Romanian and Ukrainian version of the provincial law gazette is available at the ALEX online law collection of the Austrian National Library: http://alex.onb.ac.at/cgi-content/alex?aid=lbu&datum=1910&page=118&size=45

16. 
*Bukowinisches LGBl*. 1910*,* Nr. 27, §§ 55–62, esp. § 59, pp. 125–128.

17. 
*Bukowinisches LGBl*. 1910, Nr. 26, § 31a, p. 102.

18. There is very little academic literature on the Galician Compromise, and nothing in English. For a survey article see Kuzmany (2013). For details on the circumstances leading to the Galician Compromise, see Buszko (1956), and an article of a contemporary legal scholar, Starzyński (1918). Grodziski's studies (1993, 2000) on the general history of the Galician Diet are very helpful as well.

19. There are many excellent studies (books, edited volumes and articles) on aspects of Galicia during the quasi-autonomous period, though no general study on the province's socio-political history stands out. In English, see Hann and Magocsi (2005), and Magocsi (2002). In German, see Maner (2007), and Marschall von Bieberstein (1993). In Polish, see Kieniewicz (1952) and Kawalec, Wierzbieniec, and Zaszkilniak (2011).

20. 
*Dziennik ustaw i rozporządzeń krajowych dla Królestwa Galicyi i Lodomeryi wraz z Wielkiem Księstwem Krakowskiem (Dziennik ustaw dla Galicyi). 1914. Część X. Nr. 65. Wydana i rozesłana dnia 12. lipca 1914*. *Ordynacya wyborcza*, §§ 19–21, p. 186–189. The Polish version of the provincial law gazette is available at the ALEX online law collection of the Austrian National Library: http://alex.onb.ac.at/cgi-content/alex?aid=lga&datum=1914&page=335&size=45

21. 
*Dziennik ustaw dla Galicyi*, 1914, Nr. 65, *Statut krajowy*, §§ 11, 12, p. 171.

22. Austria and Hungary both had ministers of finance within their governments. The joint Austro-Hungarian minister of finance was only responsible for the financial affairs of the federation's common tasks like the military, customs and diplomacy.

23. 
*Gesetz- und Verordnungs-Blatt für Bosnien und die Hercegovina. 1910. Nr. 19, Allerhöchste Entschließungvom 17. Februar 1910.* Landesstatut und Wahlordnung, *Landesstatut* § 22, *Wahlordnung* § 5, pp. 30–31. The German version of the provincial law gazette is available at the ALEX online law collection of the Austrian National Library: http://alex.onb.ac.at/cgi-content/alex?aid=lbh&datum=1910&page=43&size=45

24. 
*Stenographische Protokolle über die Sitzungen des Hauses der Abgeordneten des Österreichischen Reichsrates. XVIII. Session, 4. Sitzung am 27. Juni 1907* (Wien: K. K. Hof- und Staatsdruckerei), *Sitzungsprotokolle*, p. 124.
